# Tailoring the Immune Response via Customization of Pathogen Gene Expression

**DOI:** 10.1155/2014/651568

**Published:** 2014-02-25

**Authors:** Lisa M. Runco, Charles B. Stauft, J. Robert Coleman

**Affiliations:** ^1^Department of Life Sciences, New York Institute of Technology (NYIT), Old Westbury, NY 11568, USA; ^2^Codagenix, Inc., Long Island High Technology Incubator, Stony Brook, NY 11794, USA; ^3^Department of Biology, Farmingdale State College (SUNY), Farmingdale, NY 11735, USA

## Abstract

The majority of studies focused on the construction and reengineering of bacterial pathogens have mainly relied on the knocking out of virulence factors or deletion/mutation of amino acid residues to then observe the microbe's phenotype and the resulting effect on the host immune response. These knockout bacterial strains have also been proposed as vaccines to combat bacterial disease. Theoretically, knockout strains would be unable to cause disease since their virulence factors have been removed, yet they could induce a protective memory response. While knockout strains have been valuable tools to discern the role of virulence factors in host immunity and bacterial pathogenesis, they have been unable to yield clinically relevant vaccines. The advent of synthetic biology and enhanced user-directed gene customization has altered this binary process of knockout, followed by observation. Recent studies have shown that a researcher can now tailor and customize a given microbe's gene expression to produce a desired immune response. In this commentary, we highlight these studies as a new avenue for controlling the inflammatory response as well as vaccine development.

## 1. Introduction to the Codon-Pair Customization of Pathogens

The emerging field of synthetic biology and microbe design has the potential to impact many fields, including vaccine construction and the control of the host immune response [1, 2]. Viral vaccine strains with attenuated virulence using rational gene customization have been produced using computer software and *de novo* DNA synthesis allowing for genome-scale modifications to the nucleotide sequence [3–5]. Synthetic viral genes have been reengineered based on modification of the genetic phenomena of both codon bias (CB) and codon-pair bias (CPB). These two approaches can be considered synonymous customization of a target gene because they rely on synonymous codon substitutions for a given amino acid by inserting silent mutations into viral or bacterial genes rendering the amino acid sequence unchanged. Thus, target genes are “synonymously” recoded to yield customized DNA sequences. CB focuses on inserting into target genes individual, rare synonymous codons at a given position as means to control virulence and translation efficiency. For example, the rarest host codon is swapped into the target viral genome and this in turn inhibits translation. CB deoptimization was successfully applied to the capsid region of poliovirus yielding live-attenuated vaccine candidates [4, 5]. CPB is independent of CB because it normalizes for codon usage within the genome prior to calculating the representation of each codon pair, and it focuses at the level of the pairing of adjacent codons within a target gene. CPB deoptimization has also been demonstrated as a means to attenuate viral virulence (e.g., poliovirus, influenza A virus, and HIV) and construct vaccine candidates by using different synonymous codon-pairs [3, 6, 7]. Importantly both of these approaches can control viral virulence but the modified genomes still encode identical amino acids as the wild type. This allows for a perfect match to the wildtype target and, in turn, a productive immune response as a vaccine candidate. Both avenues for synonymous customization exploit redundancy in the genetic code to produce genes with altered rates of translation.

Most recently, this process of microbe design was applied beyond viruses to pathogenic bacteria [2]. In this review, we will focus on CPB modification because the studies in the bacterial pathogen *Streptococcus pneumoniae *(SP) demonstrated the ability to use CPB modification as a means to modulate the host immune response in addition to the control of gene expression [2]. Both CB and CPB have greatly expanded the sequence space for gene customization. The microbiologist is no longer bound by the binary (light switch on/off) approach of wildtype or knockout strain for bacterial gene modification. CPB modification allows for manipulation of the nucleotide sequence of a target gene, in a stepwise fashion to control its rate of expression [2, 3]. It has been demonstrated that CPB customization of a target gene allows for the titration of gene expression, akin to a light dimmer, whereby the denser the recoded gene is in rare codon-pairs, the slower it is translated and this can be controlled along a gradient. The same CPB engineering approach for viral vaccine design as in Coleman et al. and Mueller et al. [3, 16] was used to modify the toxin ofSP, pneumolysin (PLY). The resulting SP strain had reduced levels of pneumolysin gene* (ply)* expression and decreased virulence *in vivo* [2, 3]. The novelty of this approach lies in the fact that protein expression of the rationally designed gene is reduced but not eliminated. Furthermore, expression was reduced in a stepwise titration allowing for progressive reduction of virulence factor expression; the denser *ply* was in statistically underrepresented codon-pairs, the slower it was translated. Previous approaches to virulence gene regulation in bacteria relied upon knockouts of gene targets and eliminating gene expression completely, thus removal of the very antigen the immune system may need to raise a response against. As their name implies, virulence factors are gene and gene products expressed by bacterial pathogens that induce damage in the host organism and have been targets for vaccine development [11]. A virulence factor is a generic term and these disease-causing gene products include secreted toxins, pili used for host attachment, and capsules inhibiting phagocytosis. While virulence factors are possessed by any bacteria classified as pathogenic [12] and have been the initial focus of CPB modification, this gene design approach is in essence a means to slow the rate of translation of any target gene; hence it can be used for gene modification in all types of bacteria.

CPB describes a phenomenon whereby the codons that encode two sequential amino acids are found adjacent to one another with a higher or lower frequency than would be expected if codon-pairing in genes occurred randomly, without a preference [3, 6]. Similarities in codon-pairing frequency are observed across the three domains of life, although specific codon-pairs representation/preference differs from species to species [13]. CPB is observed in the genes of many organisms, including humans, and can be quantified statistically [3, 14]. CPB can influence the rate of translation [3]. Measuring and evaluating every codon-pair in the human genome while normalizing for codon usage, it was observed that some pairs occur statistically, less than if pairing was random. This suggests that there is a selective pressure to “avoid” these underrepresented pairs. The phenomenon of CPB suggests that codon-pairs which inhibit ribosome function might be “underrepresented” or avoided in expressed genes, because the corresponding tRNAs interact unfavorably within the sliding ribosome, thereby reducing the efficiency of translation [2, 15]. The specific mechanism behind how these underrepresented codon-pairs actually slow the rate of translation has yet to be illuminated but is an extremely interesting avenue to pursue so we may further understand codon-pairing preference as it relates to basic gene structure. Furthermore, underrepresented pairs have slowed translation for genes in human cells (eukaryote) and bacterial cells (prokaryote), supporting the hypothesis of their ability to slow the rate of translation across species types. In the original study of CPB customization for vaccine construction, the synthetic alteration of adjacent codon-pairs in poliovirus and influenza A virus decreased translation efficiency of the viral genomes, resulting in significant attenuation of viral virulence *in vivo* [3, 16]. The synthesized and wild-type viruses had identical amino acid sequences; however, “recoding” the viruses with synonymous, underrepresented (i.e., slow) codon-pairs resulted in a reduction in virulence [3, 17]. Interestingly, another group in Spain, using the CPB deoptimization approach, was able to phenotypically affect and alter the replicative properties of HIV-1 [7]. This demonstrates that deoptimizing the CPB of viruses to have genomes translated slowly by the host ribosome could be a universal approach for altering viral replication and pathogenicity, regardless of genome structure [17].

Building upon the studies in viral systems, CPB customization was expanded to the bacterial pathogen *Streptococcus pneumoniae* (SP), the leading cause of pneumonia globally [18]. SP was targeted for CPB modification to begin a new avenue for overcoming the emerging clinical phenomenon known as serotype replacement (STR) [19]. SP has a total of 92 serotypes; however, only 13 or 23 of these serotypes are covered in the current pediatric or adult vaccines, respectively [20]. STR is the growing emergence of SP serotypes not covered by currently utilized anti-SP vaccines, and thus these SP serotypes are becoming the major cause of pneumonia, invasive disease, and other complications from a SP infection [19]. Therefore, what is sought is a “universal” SP vaccine that provides cross-serotype protection and some in the SP field have suggested that a whole cell, live-attenuated strain might yield a “universal” SP vaccine [21]. Coleman et al.'s study focused on a STR serotype-3 SP (SP3) strain and specifically CPB modification was applied to deoptimize the pneumolysin toxin gene (*ply*) expression. By synthetically modifying *ply*, the SP3 strain constructed (SynSP3) was attenuated, as compared to both the wild type SP3 and, surprisingly, a control Δ*ply*SP3 knockout strain [2]. The initial attenuation of SP3 afforded by CPB modification was a novel advancement in bacterial vaccine design that may allow for improved *in vivo* efficacy of future vaccine candidates. CPB customization to downmodulate the expression of *ply* in SP demonstrated that we could now for the first time have strains expressing wildtype virulence factors, which are known to stimulate the immune response. For example, pneumolysin (PLY) is a known immune activator even at subhemolytic levels [22] and recently it has been shown to activate mast cells (especially at subhemolytic levels) to have increased pneumococcal clearance [23]. Mast cells activated by PLY allowed for enhanced early detection and could limit pneumococcal dissemination during invasive pulmonary pneumococcal disease. Therefore, by including wildtype virulence factors in future vaccine formulation, we can utilize strains that possess all wildtype epitopes the host needs to raise an immune response against and we hypothesize yield vaccine strains with increased *in vivo* efficacy. The nature of the immune response elicited by SynSP3 administered to experimental animals has also provided insights into the underlying mechanism of vaccine-recipients' immune response.

## 2. User-Directed Modulation of the Host Immune Response: Beyond Knockout Strains

Previously, the construction of live-attenuated bacterial vaccines has mainly focused on knocking out virulence genes to yield either a weakened strain or a strain that could serve to carry other antigens [24, 25]. This is a straight forward, logical approach: knockout (remove) the genes that cause host damage and the resulting stains will still provide protection by presenting all other protective epitopes. However, this approach has proven largely unsuccessful and there is no knockout-vaccine strain currently on the market in the US. For example, the leading causative agent of human bacterial enteric infections and bacterial diarrhea in the world is *Enterotoxigenic E. coli* (ETEC). Currently, there is no vaccine for ETEC. Annually there are over a billion BEI episodes of disease and several million deaths in developing countries [26, 27]. ETEC-induced diarrhea is the most common illness experienced by international travelers and deployed US soldiers [28]. Thus far, all antigen-based vaccines, as well as prototype knockout vaccines against human ETEC, have shown low protective efficacy [29]. Natural exposure to ETEC strains in endemic settings as well as among travelers results in the development of protective immunity, indicating a live-attenuated vaccine capable of stimulating the adaptive immune response that would provide long-term immunity [27, 30]. Knockout-ETEC strains have been entered into clinical trials [31, 32] to test as vaccines, yet they have been unsuccessful [29]. It is our hypothesis that knockout bacterial strains for many bacterial diseases will have difficulty ever becoming live-attenuated vaccines because by knocking out virulence factors, a “too cold” immune response is induced in the recipient, preventing the induction of protective immunity [2]. There are multiple examples of attenuated bacterial knockout strains that were tested as vaccines in humans and/or experimental animals [29, 33, 34]; while these strains have been valuable in demonstrating the role of these virulence factors in pathogenesis, their inability to function as commercial vaccines highlight the drawbacks associated with virulence factor knockout. The initial data showing decreased toxin expression and *in vivo* efficacy by the synthetic SP stain has demonstrated that the “re-coding” of bacterial toxin genes is an innovative new avenue for bacterial vaccine construction.

**Figure 1:**
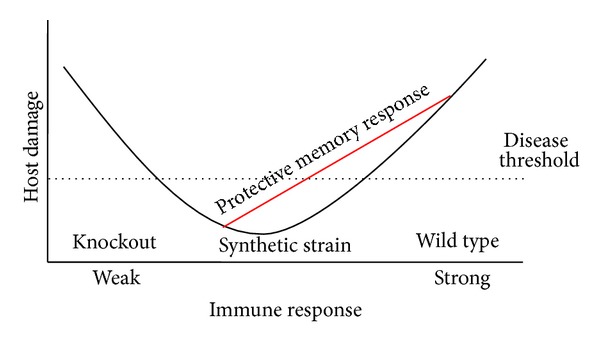
Induction of a “just right” immune response, as explained with regard to the damage-response framework (DRF). This curve, based on the DRF of how a host responds to virulence factors and pathogens, was first put forth by Pirofski and Casadevall [8, 9]. The DRF hypothesizes that human disease resulting from microbial infection manifests in two ways: (1) there is a response that is too robust (wild type), which is deleterious to the host or (2) a response that is insufficient, allowing unchecked invasion and succumbing to infection. Also, the low immune response portion of the curve can be viewed with regard to knockout strains with low efficacy as vaccines. Synthetic strains would be in the minimum of the DRF because they are expressing low levels of the toxin, allowing for an increased immune response as compared to the knockout, but below the disease threshold.

**Figure 2:**
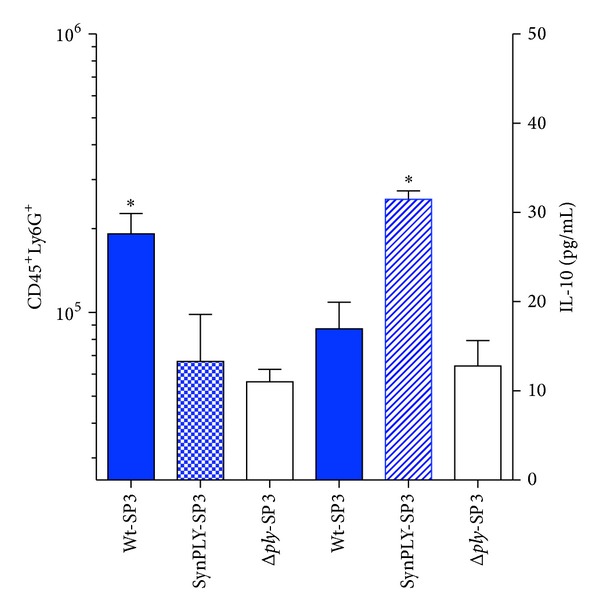
Modulation of the immune response by using codon-pair bias to titrate bacterial toxin expression. Codon-pair bias customization provides for the titration of expression and modulation of the host immune response. By reducing *Streptococcus pneumoniae* (SP) toxin expression (pneumolysin gene,* ply*) a more controlled, nondeleterious response is induced. The shading of the triangles is supported by data from Coleman et al., whereby *ply *expression is significantly reduced but not eliminated [2]. The wildtype serotype-3 SP (Wt-SP3) expresses 8 hemolytic units (HU) per mL of supernatant from an eight-hour growing culture, whereas SynSP3 produces 2 HU/mL and Δ*ply*-SP3 produces 0 HU/mL (data not shown). The infection of mice with Wt-SP3, which secretes high levels of pneumolysin, induces an excessive, deleterious recruitment of CD45+Ly6G+ neutrophils (PMNs) to the lungs 48 hours postinfection, a known manifestation of pulmonary pneumococcal disease [10]. The left *y*-axis is log_10_ scale and corresponds to the total number of PMNs isolated from lungs of mice infected with the indicated strains. Interestingly Δ*ply*-SP3 recruited the fewest PMNs and SynSP3 an intermediary quantity, corresponding to the hypothesis of the DRF of “just right” immune response. The right *y*-axis is a linear axis and corresponds to the quantity of IL-10 isolated via ELISA from lungs of mice infected with the indicated strain (right axis). IL-10 is an anti-inflammatory cytokine, so the increased expression of IL-10 in the lungs of SynSP3-infected mice may allow for the “controlled” nondeleterious PMN infiltration. The SynSP3-infected mice recruit fewer PMNs (left axis) and possess the highest level of IL-10 (right axis). The control Δ*ply* knockout strain secretes no PLY and fails to stimulate the immune response, with the least PMNs (left) and IL-10 levels (right axis). The graphs have been placed on a single figure for the ease of comparison and not to read each *y*-axis simultaneously.

The findings provided by the CPB-modified SP3 strain may shed light onto why this knockout approach has been unsuccessful and provide a new method for constructing live-attenuated bacterial vaccines. Virulence gene knockout is a binary approach; the virulence factor is either present at wild type levels or completely removed. In contrast, expression using CPB deoptimization can be used to *progressively* dampen expression of genes [2]. No longer is the researcher limited to a binary control over bacterial virulence gene expression (wild type versus knockout). It now seems that the expression of virulence genes may be required for immune system activation and response [23, 35, 36]. At wild type levels, these virulence genes serve to damage the host, causing disease and possibly death. When virulence genes are knocked out in the host, a muted immune activation may occur and the host may fail to mount a full and proper response (possibly a reason previous knockout strains were inadequate as vaccines) [12, 23, 31]. The damage-response framework (DRF) of how a host responds to virulence factors and pathogens was first put forth by Pirofski and Casadevall [8, 9]. The DRF hypothesizes that human disease resulting from microbial infection manifests in two ways—either there is a response that is too robust (which is deleterious to the host) or a response that is insufficient, allowing unchecked invasion and in turn succumbing to infection. Pertinent examples of these two outcomes are (1) the “too hot” immune response known as the “cytokine storm,” which is thought to be one of the main causes of death by the 1918 influenza [37], or (2) the “too cold” immune response, allowing for evasion by certain SP serotypes (e.g., serotype 8 SP) that express nonhemolytic pneumolysin. These nonhemolytic serotypes are the major cause of invasive pneumococcal disease due to a lack of immune induction [38, 39]. Only two points on the DRF curve could be obtained prior to Coleman et al., the “too hot” or “too cold” immune response to pathogens. CPB customization, which provides for *progressive* downregulation of expression of the toxin *ply* in SP3, began to provide the first evidence to support the DRF because it provides intermediate points on the curve. Specifically, *progressive* downregulation of a virulence factor allowed for the induction of the “just right” immune response preventing disease in infected animals and inducing protective immunity (Figure 1). In Figure 1, the DRF curve has been adapted to demonstrate the “just right” immune response elicited by a theoretically synthetic strain like SynSP3—there is minimal host damage but maximal protective immune response. There is experimental evidence to support this hypothesis. When the expression of *ply* was significantly reduced in SynSP3, but not eliminated, virulence was reduced and this was a function of the type of immune response elicited [2]. Mice infected with the wild type SP3 strain experienced an overexuberant (uncontrolled) inflammatory response caused by wild type levels of the toxin pneumolysin. This response was characterized by lower levels of CD4^+^ and CD8^+^ cells as compared to SynSP3 [2], as well as an excessive and deleterious recruitment to the lungs of CD45^+^LY6G^+^ polymorphonuclear leukocytes (PMNs) whose bacterial clearance function is inhibited by pneumolysin (Figure 2) [40]. It is known that the deleterious immune response caused by wild type SP3 is characterized by significant (and excessive) IL-17 and other proinflammatory cytokine secretions [41]. Additionally, a disproportionate cellular response occurs that is weighted towards PMNs, which cause excessive host damage [42, 43]. Mice infected with the wild type SP3 secreted less of the cytokine IL-10 in comparison to mice infected with SynSP3 (Figure 2). IL-10 is a known anti-inflammatory cytokine and regulator of the inflammatory response. Elevated levels of IL-10 cytokine have been correlated with increased survival in SP-infected mice [44]. These findings by Coleman et al. supported the hypothesis that wild type SP3 causes an overexuberant, “too hot” inflammatory response that was insufficiently regulated due to an inadequate expression of IL-10. In contrast, mice infected with a Δ*ply*SP3 strain produced a “too cold” immune response that could be characterized as underwhelming, when observing PMN (Figure 2) as well as CD4^+^ and CD8^+^ cells (data not shown) infiltration [2]. Mice infected with a Δ*ply*SP3 strain had a muted recruitment of immune cells and succumbed to infection at an equal rate as wild type SP3 [2]. The wild type SP3 strain and the Δ*ply*SP3 represent the previous binary approach to virulence factor engineering and vaccine construction, demonstrating the over- or underwhelming immune response induced by each, and lastly, that the Δ*ply*SP3 knockout strain could not serve as a vaccine. However, the SynSP3 that induced the “just right” immune response allowed for a controlled inflammation (as inferred by the expression of IL-10) and allowed for clearance of the bacteria and the induction of a memory response.

We see that infection of mice with SynSP3 is not associated with an overexuberant recruitment of PMNs to the lungs although upregulation of the immune modulator IL-10 is observed (Figure 2). SynSP3 recruited the highest number of CD19^+^ B cells to the lungs of infected mice as compared to the other strains and we hypothesize that these CD19^+^ B cells could be the source of the IL-10 [45]. Also, mice infected with SynSP3 recruited the highest levels of CD4^+^ and CD8^+^ T cells *in vivo* and were able to activate dendritic cells (DCs) when cocultured *in vitro* [2]. Activated antigen presenting cells like DCs are a known correlate of protection induced by vaccination [46]. By using genetic modification to control virulence factor expression so that the immune response is not “too hot” (wild type) and/or not “too cold” (Δ*ply*SP3), but rather “just right” (SynSP3) may allow for the construction of other live-attenuated vaccines capable of a robust cellular immune response.

In viral systems, it is thought that a protective immune response to influenza is a controlled, nondeleterious pulmonary inflammation that limits excessive tissue damage. More specifically, the IL-10 production is thought to prevent this overexuberant inflammatory response to an influenza infection [47]. Thus, a live-attenuated influenza vaccine should also allow for IL-10 expression while replicating following vaccination. Recent studies have suggested the source of IL-10 in the context of an influenza infection may be CD4^+^ and CD8^+^ T cells, and IL-10 expression is a high correlate to survival/protection against influenza [47]. Coleman et al. also found the highest levels of CD8^+^ T cells in the lungs of SynSP3-infected mice. CD8^+^ T cells are also required for survival of an SP3 infection and could be the source of this IL-10 as well [41]. Furthermore, IL-10 is such a potent regulator of overexuberant immune responses that there has been a human clinical trial of a commensal bacterial strain *Lactococcus lactis* expressing IL-10 to combat Crohn's disease [48]. While here we only highlight the induction of increased IL-10 and controlled PMN tissue infiltration by a synthetically-modified strain expressing the “just right” level of immune stimulating toxin, it is our hypothesis that genetically designed strains that can induce an enhanced, nondeleterious immune response represent a brand new avenue for vaccine development to combat many pathogens currently affecting human health. A vaccine that mimics a natural infection (i.e., live attenuated) will elicit the most robust response. The user-directed design and titration of virulence factor expression will not only open a new avenue for vaccine development but also provide basic insights into the effects of codon pairing on bacterial gene expression.

## 3. Conclusion

The CPB-modulation of genes that relies on DNA synthesis and user-directed design allows for a more fine-tuned control of expression as compared to gene knockout and other conventional genetic modifications [2, 5, 6]. Additionally these findings have begun to highlight the role of the inflammatory response in the setting of bacterial infection. Specifically there may be a possible yin-yang with regard to virulence factors and their interaction with the immune system, providing support for the DRF [9, 12]. In sum, lower doses of virulence factors produced by synthetically-modified genes could be beneficial because they stimulate immunity enough to induce pathogen clearance but not enough to cause hyperinflammation and ultimately host morbidity and mortality. These results may be a significant finding because the application of gene customization to the construction of microbes with attenuated virulence is relevant and important to the microbial pathogenesis and vaccine development fields.

## References

[1] Mueller S, Coleman JR, Wimmer E (2009). Putting synthesis into biology: a viral view of genetic engineering through de novo gene and genome synthesis. *Chemistry and Biology*.

[2] Coleman JR, Papamichail D, Yano M, Del Mar García-Suárez M, Pirofski L-A (2011). Designed reduction of Streptococcus pneumoniae pathogenicity via synthetic changes in virulence factor codon-pair bias. *Journal of Infectious Diseases*.

[3] Coleman JR, Papamichail D, Skiena S, Futcher B, Wimmer E, Mueller S (2008). Virus attenuation by genome-scale changes in codon pair bias. *Science*.

[4] Mueller S, Papamichail D, Coleman JR, Skiena S, Wimmer E (2006). Reduction of the rate of poliovirus protein synthesis through large-scale codon deoptimization causes attenuation of viral virulence by lowering specific infectivity. *Journal of Virology*.

[5] Burns CC, Shaw J, Campagnoli R (2006). Modulation of poliovirus replicative fitness in HeLa cells by deoptimization of synonymous codon usage in the capsid region. *Journal of Virology*.

[6] Gutman GA, Hatfield GW (1989). Nonrandom utilization of codon pairs in *Escherichia coli*. *Proceedings of the National Academy of Sciences of the United States of America*.

[7] Martrus G, Nevot M, Andres C, Clotet B, Martinez MA (2013). Changes in codon-pair bias of human immunodeficiency virus type 1 have profound effects on virus replication in cell culture. *Retrovirology*.

[8] Casadevall A, Pirofski L-A (2003). The damage-response framework of microbial pathogenesis. *Nature Reviews. Microbiology*.

[9] Pirofski L-A, Casadevall A (2008). The damage-response framework of microbial pathogenesis and infectious diseases. *Advances in Experimental Medicine and Biology*.

[10] Tian H, Groner A, Boes M, Pirofski L-A (2007). Pneumococcal capsular polysaccharide vaccine-mediated protection against serotype 3 *Streptococcus pneumoniae* in immunodeficient mice. *Infection and Immunity*.

[11] Kaufmann SHE (2007). The contribution of immunology to the rational design of novel antibacterial vaccines. *Nature Reviews Microbiology*.

[12] Casadevall A, Pirofski L-A (2009). Virulence factors and their mechanisms of action: the view from a damage-response framework. *Journal of Water and Health*.

[13] Tats A, Tenson T, Remm M (2008). Preferred and avoided codon pairs in three domains of life. *BMC Genomics*.

[14] Moura G, Pinheiro M, Arrais J (2007). Large scale comparative codon-pair context analysis unveils general rules that fine-tune evolution of mRNA primary structure. *PLoS ONE*.

[15] Fedorov A, Saxonov S, Gilbert W (2002). Regularities of context-dependent codon bias in eukaryotic genes. *Nucleic Acids Research*.

[16] Mueller S, Coleman JR, Papamichail D (2010). Live attenuated influenza virus vaccines by computer-aided rational design. *Nature Biotechnology*.

[17] Coffin JM (2008). Attenuation by a thousand cuts. *The New England Journal of Medicine*.

[18] Weinberger DM, Malley R, Lipsitch M (2011). Serotype replacement in disease after pneumococcal vaccination. *The Lancet*.

[19] Hicks LA, Harrison LH, Flannery B (2007). Incidence of pneumococcal disease due to non- pneumococcal conjugate vaccine (PCV7) serotypes in the United States during the era of widespread PCV7 vaccination, 1998–2004. *Journal of Infectious Diseases*.

[20] MMWR (2005). Direct and indirect effects of routine vaccination of children with 7-valent pneumococcal conjugate vaccine on incidence of invasive pneumococcal disease—United States, 1998–2003. *Morbidity and Mortality Weekly Report*.

[21] Roche AM, King SJ, Weiser JN (2007). Live attenuated Streptococcus pneumoniae strains induce serotype-independent mucosal and systemic protection in mice. *Infection and Immunity*.

[22] Rubins JB, Charboneau D, Paton JC, Mitchell TJ, Andrew PW, Janoff EN (1995). Dual function of pneumolysin in the early pathogenesis of murine pneumococcal pneumonia. *Journal of Clinical Investigation*.

[23] Cruse G, Fernandes VE, De Salort J (2010). Human lung mast cells mediate pneumococcal cell death in response to activation by pneumolysin. *Journal of Immunology*.

[24] Clemens J, Savarino S, Abu-Elyazeed R (2004). Development of pathogenicity-driven definitions of outcomes for a field trial of a killed oral vaccine against enterotoxigenic *Escherichia coli* in Egypt: application of an evidence-based method. *Journal of Infectious Diseases*.

[25] Mitchell TJ, Walker JA, Saunders FK, Andrew PW, Boulnois GJ (1989). Expression of the pneumolysin gene in *Escherichia coli*: rapid purification and biological properties. *Biochimica et Biophysica Acta*.

[26] Nataro JP, Kaper JB (1998). Diarrheagenic *Escherichia coli*. *Clinical Microbiology Reviews*.

[27] Gupta SK, Keck J, Ram PK, Crump JA, Miller MA, Mintz ED (2008). Part III. Analysis of data gaps pertaining to Enterotoxigenic *Escherichia coli* infections in low and medium human development index countries, 1984–2005. *Epidemiology and Infection*.

[28] Rockabrand DM, Shaheen HI, Khalil SB (2006). Enterotoxigenic *Escherichia coli* colonization factor types collected from 1997 to 2001 in US military personnel during operation Bright Star in northern Egypt. *Diagnostic Microbiology and Infectious Disease*.

[29] Qadri F, Ahmed T, Ahmed F (2003). Safety and immunogenicity of an oral, inactivated enterotoxigenic *Escherichia coli* plus cholera toxin B subunit vaccine in Bangladeshi children 18–36 months of age. *Vaccine*.

[30] Levine MM, Nalin DR, Hoover DL (1979). Immunity to enterotoxigenic *Escherichia coli*. *Infection and Immunity*.

[31] Darsley MJ, Chakrabortyb S, DeNearing B The oral, live attenuated enterotoxigenic *Escherichia coli* vaccine ACE527 reduces the incidence and severity of diarrhea in a human challenge model of diarrheal disease. *Clinical and Vaccine Immunology*.

[32] Steinsland H, Valentiner-Branth P, Aaby P, Mølbak K, Sommerfelt H (2004). Clonal relatedness of enterotoxigenic *Escherichia coli* strains isolated from a cohort of young children in Guinea-Bissau. *Journal of Clinical Microbiology*.

[33] Skeiky YAW, Sadoff JC (2006). Advances in tuberculosis vaccine strategies. *Nature Reviews Microbiology*.

[34] García V, Gómez M, Iglesias M (1996). Intramammary immunization with live-attenuated *Staphylococcus aureus*: microbiological and immunological studies in a mouse mastitis model. *FEMS Immunology and Medical Microbiology*.

[35] McNeela EA, Burke Á, Neill DR (2010). Pneumolysin activates the NLRP3 inflammasome and promotes proinflammatory cytokines independently of TLR4. *PLoS Pathogens*.

[36] Kulkarni R, Dhakal BK, Slechta ES, Kurtz Z, Mulvey MA, Thanassi DG (2009). Roles of putative type II secretion and type IV pilus systems in the virulence of uropathogenic *Escherichia coli*. *PLoS ONE*.

[37] Morens DM, Fauci AS (2007). The 1918 influenza pandemic: insights for the 21st century. *Journal of Infectious Diseases*.

[38] Jefferies JMC, Johnston CHG, Kirkham L-AS (2007). Presence of nonhemolytic pneumolysin in serotypes of *Streptococcus pneumoniae* associated with disease outbreaks. *Journal of Infectious Diseases*.

[39] Marks M, Burns T, Abadi M (2007). Influence of neutropenia on the course of serotype 8 pneumococcal pneumonia in mice. *Infection and Immunity*.

[40] Paton JC, Ferrante A (1983). Inhibition of human polymorphonuclear leukocyte respiratory burst, bactericidal activity, and migration by pneumolysin. *Infection and Immunity*.

[41] Weber SE, Tian H, Pirofski L-A (2011). CD8+ cells enhance resistance to pulmonary serotype 3 *Streptococcus pneumoniae* infection in mice. *Journal of Immunology*.

[42] Moreland JG, Bailey G (2006). Neutrophil transendothelial migration in vitro to Streptococcus pneumoniae is pneumolysin dependent. *American Journal of Physiology: Lung Cellular and Molecular Physiology*.

[43] Craig A, Mai J, Cai S, Jeyaseelan S (2009). Neutrophil recruitment to the lungs during bacterial pneumonia. *Infection and Immunity*.

[44] Wang E, Bergeron Y, Bergeron MG (2005). Ceftriaxone pharmacokinetics in interleukin-10-treated murine pneumococcal pneumonia. *Journal of Antimicrobial Chemotherapy*.

[45] Yanaba K, Bouaziz J-D, Haas KM, Poe JC, Fujimoto M, Tedder TF (2008). A regulatory B cell subset with a unique CD1dhiCD5+ phenotype controls T cell-dependent inflammatory responses. *Immunity*.

[46] Plotkin SA (2001). Immunologic correlates of protection induced by vaccination. *Pediatric Infectious Disease Journal*.

[47] Sun J, Madan R, Karp CL, Braciale TJ (2009). Effector T cells control lung inflammation during acute influenza virus infection by producing IL-10. *Nature Medicine*.

[48] Braat H, Rottiers P, Hommes DW A phase I trial with transgenic bacteria expressing interleukin-10 in Crohn’s disease. *Clinical Gastroenterology and Hepatology*.

